# Association between chronic *Anaplasma marginale* and *Babesia* spp. infection and hematological parameters of taurine heifers

**DOI:** 10.1590/S1984-29612023052

**Published:** 2023-09-01

**Authors:** Natalia Machado Rahal, Gabriela Bueno Luz, Kauê Rodriguez Martins, Bernardo Garziera Gasperin, Josiane de Oliveira Feijó, André Gustavo Cabrera Dalto, Monique Tomazele Rovani, Rodrigo Casquero Cunha, Marcio Nunes Corrêa

**Affiliations:** 1 Núcleo de Pesquisa, Ensino e Extensão em Pecuária – NUPEEC, Faculdade de Veterinária, Universidade Federal de Pelotas – UFPel, Pelotas, RS, Brasil; 2 Laboratório de Biologia Molecular Veterinária – LaBMol-Vet, Faculdade de Veterinária, Universidade Federal de Pelotas – UFPel, Pelotas, RS, Brasil; 3 Rede Fibra, Faculdade de Veterinária, Universidade Federal de Pelotas – UFPel, RS, Brasil; 4 Setor de Grandes Ruminantes, Faculdade de Veterinária, Universidade Federal do Rio Grande do Sul – UFRGS, Porto Alegre, RS, Brasil

**Keywords:** Cattle herd, timed artificial insemination, cattle tick fever, qPCR, Rebanho bovino, inseminação artificial em tempo fixo, tristeza parasitária bovina, qPCR

## Abstract

The aim of this study was to investigate the association between chronic *Anaplasma marginale* and *Babesia* spp. infection and hematological parameters of pregnant and non-pregnant taurine heifers. Blood samples from 94 females were collected on the first day (D-10) of timed artificial insemination (TAI) protocol and on pregnancy diagnosis (D+34). Hematological parameters were determined and compared between pregnant (PG) and non-pregnant (NPG) heifers, and within group at different sampling days. Real-time PCR (qPCR) was used to determine *A. marginale* and *Babesia bovis* infection, and for absolute quantification of *Babesia* spp. between PG and NPG groups. Correlation analysis was performed between the number of gDNA copies (CN) of *Babesia* spp. and hematological parameters. On D-10, mean hemoglobin concentration was higher for NPG, and hematocrit and total plasma protein were higher on D+34 for both groups. There was no difference in *Babesia* spp. CN between groups. In the first qPCR, all heifers were positive for *A. marginale* and *B. bovis*. Significant correlations were found between hemoglobin and erythrocyte and between hemoglobin and hematocrit (r = 0.8082 and r = 0.3009, respectively). Low levels of *A. marginale* and *Babesia* spp. did not affect hematological parameters of chronically infected pregnant and non-pregnant taurine heifers.

## Resumo

O objetivo deste estudo foi investigar a associação entre infecção crônica por *Anaplasma marginale* e *Babesia* spp. e parâmetros hematológicos de novilhas taurinas prenhes e não prenhes. Sangue de 94 fêmeas foi coletado no primeiro dia (D-10) do protocolo de inseminação artificial em tempo fixo (IATF) e no diagnóstico de gestação (D+34). Parâmetros hematológicos foram comparados entre novilhas prenhes (PG) e não prenhes (NPG) e dentro dos grupos entre dias de coleta. Usando-se PCR em tempo real (qPCR), determinou-se a infecção por *A. marginale* e *Babesia bovis* e quantificação absoluta de *Babesia* spp. Entre os grupos PG e NPG. A análise de correlação foi realizada entre o número de cópias (CN) de *Babesia* spp. e parâmetros hematológicos. No D-10, a concentração de hemoglobina foi maior para NPG e hematócrito, e proteína plasmática total foram maiores em D+34 para ambos os grupos. Não houve diferença para CN de *Babesia* spp. entre os grupos. Na primeira qPCR, todas as novilhas foram positivas para *A. marginale* e *B. bovis.* Correlações significativas foram encontradas entre hemoglobina/eritrócito e hemoglobina/hematócrito (r=0,8082 e r=0,3009, respectivamente). Baixos níveis de *A. marginale* e *Babesia* spp. não afetaram os parâmetros hematológicos de novilhas taurinas prenhes e não prenhes cronicamente infectadas.

## Introduction

Infectious tick-borne diseases can directly and indirectly affect the productive and reproductive performances of ruminants. In Brazil, one of the diseases that impact livestock is cattle tick fever (TF), a complex caused by *Babesia bovis*, *Babesia bigemina* and/or *Anaplasma marginale*, which significantly impacts herds in southern Brazil (Lucena et al., 2010) and cause major financial losses for farmers throughout the country (Barbieri et al., 2016).

Its occurrence is directly related to the distribution of the cattle tick, *Rhipicephalus* (*Boophilus*) *microplus*, and its estimated economic impact in Brazil is US$ 3.24 billion, attributable to production losses (Grisi et al., 2014). Although *R. microplus* is the primary vector for these agents, tabanids and iatrogenic transmission may also be responsible for transmission of this disease (Kocan et al., 2010; Palmer & Clothier, 2015; Zaugg, 2015). Animals from tick-free or enzootic instability areas are highly susceptible to these infections and have high morbidity rates, presenting anemia, fever, jaundice, and weight loss, and even culminating in death (Kocan et al., 2010). Also, it is known that severe anemia caused by TF results in hypoxia, which can lead to tissue hypoxia and subsequent abortion (Givens & Marley, 2008). Additionally, transplacental infection by *Babesia* spp. and *A. marginale* directly affects fetal organs, causing splenomegaly, hemorrhages, and neurological damage, and resulting in fetal loss, stillbirth, and neonatal death (Costa et al., 2016; Henker et al., 2020). Animals recovering from the acute phase of TF remain persistently infected, with low levels of parasitemia, thus perpetuating the presence of infectious agents in the herd (Kocan et al., 2010).

Over recent years, some studies using quantitative molecular techniques have been conducted. Real-time PCR (qPCR) is a more sensitive diagnostic tool that is capable of providing specific data on the number of DNA copies of parasites in different samples. Through these detailed data, it is possible to analyze the relationship between the intensity of host infection and the number of vectors or their infection intensity; also, how cattle health is affected according to fluctuations of the number of DNA copies (Martins et al., 2020; Giglioti et al., 2021; Garcia et al., 2022).

As stated above, consequences of clinical TF are well known and documented, however there are opportunities to investigate how chronic infection can affect cattle. Thus, we aimed to verify the impact of chronic TF infection on hematological parameters, using taurine heifers selected to a timed artificial insemination (TAI) protocol, and molecular techniques to diagnose *A. marginale* and *Babesia* spp.

## Material and Methods

### Animals, reproductive management, and pregnancy diagnosis

Angus heifers (n = 94) were selected for an estrus synchronization protocol in November 2019. The females were maintained in an extensive system of native pasture, located in the municipality of Charqueadas, Rio Grande do Sul, Brazil (29°58'42.1" S; 51°33'28.2" W). The inclusion criteria for TAI were age (≥ 24 months), weight (65% of the adult weight for the breed), and body condition score [BCS (≥ 2.5)]. The heifers selected had an average weight of 357.8 kg ± 25.4 and median BCS of 3 [max: 4; min: 2; scale from 1 (thin) to 5 (obese)]. During animal handling, vaginal mucosal color was assessed and classified (pale, pink, hyperemic or icteric) by the same examiner.

The heifers were subjected to a synchronization protocol using an intravaginal progesterone-releasing device (1.25 g Biprogest^®^, Bimeda, Brazil) along with i.m. administration of 2 mg of estradiol benzoate (EB; Sincroben^®^, Bimeda, Brazil) 10 days before TAI (D-10). The device was removed on D-2 and sodium cloprostenol (526 μg; Clocio^®^, Bimeda, Brazil) was administered i.m. On D-1, EB (1 mg; Sincroben^®^, Bimeda, Brazil) was injected i.m.; and on day 0 (D0) TAI was performed. Pregnancy diagnosis was performed using ultrasound on day 34 (D+34). After handling and sampling on D-10, the heifers were treated with Colosso FC30 (Ourofino Saude Animal, Brazil) using an immersion bath, as part of the farm’s procedure.

### Sample collection and processing

On D-10 and D+34, blood samples were collected from all animals by means of coccygeal vein puncture, into 4 mL vacuum tubes with EDTA, and were immediately placed in a cool box and then transported to the laboratory within 12 hours. Subsequently, the samples were used to determine hematocrit, using capillary tubes, with centrifugation for 5 min at 2,650 × g in a digital microhematocrit centrifuge (Benfer, São Paulo, Brazil). The results were determined using a microhematocrit reader, and total plasma protein was read using a portable refractometer (ITREF200, Instrutemp, Sao Paulo, Brazil). An automatic hematology analyzer (BC-2800Vet, Mindray, Brazil) was used to evaluate erythrocyte count and hemoglobin concentration.

For direct parasite detection, blood smears were made from all samples and were stained with Panotico Rapido (LaborClin, Paraná, Brazil). They were viewed under a microscope (Nikon Eclipse E200, Nikon Instruments Inc., Tokyo, Japan). In addition, a blood aliquot of 1 mL was separated from the EDTA tube, placed in a 1.5 mL microtube, and stored at -20 °C for subsequent DNA extraction and PCR analysis.

### Genomic DNA extraction and real-time PCR (qPCR)

Total genomic DNA (gDNA) was extracted from D-10 samples, using the ReliaPrep™ Blood gDNA Miniprep System (Promega, USA), in accordance with the manufacturer’s instructions. Sample DNA quantity and purity (260/280 nm ratio) were evaluated through spectrophotometry using NanoDrop™ (Thermo Fisher Scientific, USA). Initially, standard two-step real-time PCR (qPCR) was performed using GoTaq qPCR Master Mix (Promega) containing an intercalating fluorophore for *A. marginale* (F - 5' – AAGGCGAGGAGCTTTATTAAG – 3'; R - 5' - CTACTGCCTCACAAGGACGA - 3') and *B. bovis* (F - 5' - TGTTCCTGGAAGCGTTGATTC - 3'; R – 5' – AGCGTGAAAATAACGCATTGC – 3'), using primers described by Bacanelli et al. (2014) and Buling et al. (2007), respectively. The final well volume was 20 μL, and the following procedure was implemented: initial denaturation at 95 °C for 5 min, followed by 40 cycles of denaturation at 95 °C for 15 sec and annealing/extension at 58 °C for 30 sec. To ensure viability of the extracted gDNA, qPCR was also performed based on the endogenous mammalian *GAPDH* (glyceraldehyde-3-phosphate dehydrogenase) gene (F – 5’ – GATTGTCAGCAATGCCTCCT – 3’; R – 5’ – GGTCATAAGTCCCTCCACGA – 3’; Rovani et al., 2017). Melting curves were verified to ensure the identity of the result and the amplicons were sequenced and subjected to homology analysis using the BLAST software (all with required similarities greater than 96% for target genes). Samples were analyzed in duplicate, using the StepOne Plus real-time PCR system (Thermo Fisher Scientific). Samples positive for *B. bovis* in real-time PCR were quantified for *B. bigemina* and *B. bovis*.

### Absolute quantification of Babesia spp.

To determine *B. bigemina* and *B. bovis* infection levels, species-specific primers (Buling et al., 2007) and standard curves (Giglioti et al., 2016, 2017) targeting the mitochondrial cytochrome b gene for *B. bigemina* (*cBisg*) and *B. bovis* (*cBosg*) were used (Table 1).

**Table 1:** Primers and probes used for qPCR for absolute quantification of *Babesia bigemina* and *B. bovis.*

Type (5’ – 3’)	*Babesia bigemina*	*Babesia bovis*
Forward primer	TGTTCCAGGAGATGTTGATTC	GGATTGTGGTACTCAAGCAGATA
Probe	/56-FAMQCGAGTGTGT/ZEN/TATCAGAGTATTAACTGAGGT/3IABkFQ/	/56-FAMQACCATGGTC/ZEN/ATGGTATTCTGGAATGGT/3IABkFQ/
Reverse primer	AGCATGGAAATAACGAAGTGC	CCGTAAGGAAGAACATAACCTAAGA

Quantification of DNA levels was carried in the CFX Opus 96™ real-time PCR device and the data from this were obtained through the Bio-Rad CFX Maestro 2.3 software (Bio-Rad Laboratories). The final well volume was 10 µL, using 5 mL of iTaq, 0.5 µL of the F primer (10 mM), 0.5 µL of the R primer (10 mM), 2 µL of sterile ultra-pure water (Nuclease-Free Water, Promega^®^, São Paulo, Brazil) and 2 µL of the sample, diluted to a final DNA concentration of 50 ng/µL. The run method comprised an activation cycle of iTaq DNA polymerase at 95 °C for 10 min, followed by 40 cycles of denaturation at 95 °C for 45 s and an annealing/extension cycle at 60 °C for 1 min.

Using starting quantity (SQ) data from the software of the equipment, the number of DNA copies (NC) was calculated as described by Ke et al. (2006): *CN = [6.022* × *10^23^ (copies/mol)* × *concentration (g/mol)]* / *molecular mass (g/L)*, where 6.022 × 10^23^ is Avogadro’s constant, and the molecular mass is the average molecular weight of double-stranded DNA (330 × 2) multiplied by the size of the cloned fragment (Ke et al., 2006).

The calibration curve was constructed using gBlocks^®^ sequences (Integrated DNA Technologies, Inc., Coralville, IA, USA) for the cytochrome b gene for *B. bigemina* and *B. bovis*. A preliminary test was performed using serial dilutions ranging from 10^-1^ to 10^-15^, a positive control sample and randomly selected samples from the heifers. Through the initial tests, the optimal standard curves were found to range from 10^-7^ to 10^-13^ ng/µL for *B. bigemina*, and from 10^-3^ to 10^-9^ ng/µL for *B. bovis* (Supplementary file 1). Samples from one heifer infected with *B. bigemina* and one with *B. bovis*, which had previously been confirmed through blood smears and conventional PCR, as described by Buling et al. (2007), were used as positive controls, while the negative control was nuclease-free water. All the samples, including controls and gBlocks^®^, were analyzed in duplicate and samples that differed regarding the duplicate (standard deviation of the cycle quantification > 0.5) were re-analyzed. The guidelines of the "Minimum Information for Publication of Quantitative Real-Time PCR Experiments" (MIQE) (Bustin et al., 2009) were followed.

### Statistical analysis

The conception rate was calculated by dividing the number of pregnant heifers by the number of inseminated heifers (94), and multiplying by 100. The data were analyzed comparing pregnant (PG) and non-pregnant (NPG) groups, according to heifers’ pregnancy outcome on D+34. Data normality and homoscedasticity were verified through Shapiro-Wilk’s and Levene’s test, respectively. For hematocrit, total plasma protein and hematological parameters, Tukey’s test was used to compare means between groups and for pairwise comparison between sampling time (D-10/D+34) within group. Wilcoxon’s test was used for the number of gDNA copies (CN) of *Babesia* spp., and Fisher’s exact test for vaginal mucosal color.

According to the results of the normality test, Spearman’s test was used to analyze the relationship between CN of *B. bigemina* and *B. bovis* and hematological parameters. The correlation between the variables hematocrit, erythrocyte and hemoglobin, Pearson’s test was used. All correlation analyses were performed regardless of pregnancy outcome. The statistical analyses were performed using JMP Pro 14 (SAS Institute Inc.). Significance was achieved when *P* < 0.05 and a tendency when 0.05 < *P* < 0.1.

## Results and Discussion

Conception rate was 47.87% (45 pregnant and 49 non-pregnant out of 94 heifers in total), as expected for this breed and category (Sánchez-Castro et al., 2020). There was no difference between PG and NPG heifers regarding the color of the vaginal mucosa, and none of the animals presented pale or icteric mucosa. Also, as expected, due to the absence of animals with clinical signs for TF, neither *A. marginale* nor *Babesia* spp. was viewed in microscopic evaluations of blood smears.

**Figure 1:**
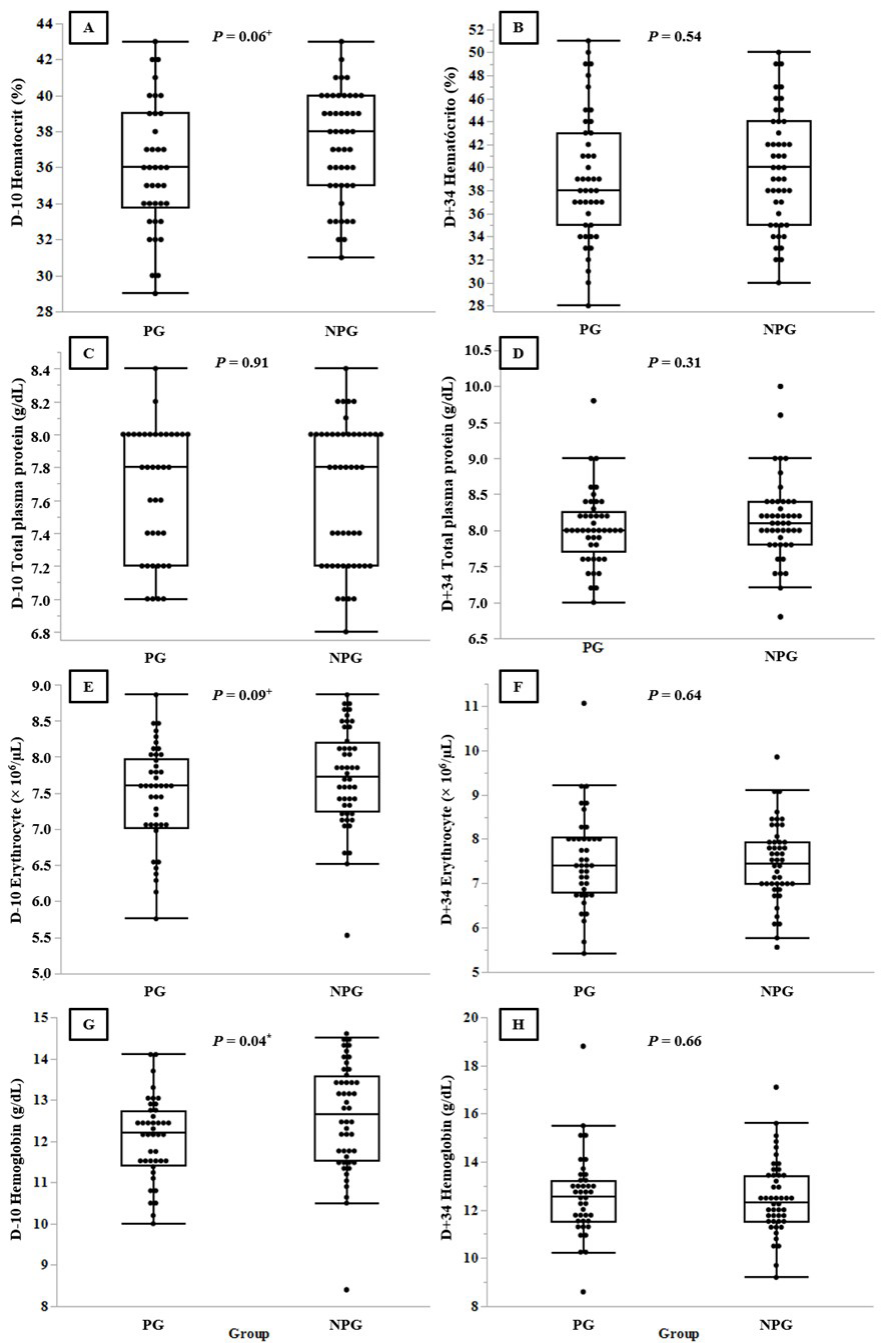
Hematological parameters of Angus heifers in the pregnant group (PG) and non-pregnant group (NPG) on the first day (D-10) of the timed artificial insemination protocol (TAI) and 34 days after the day of insemination. Hematocrit was observed on D-10 (A) and D+34 (B), total plasma protein on D-10 (C) and D+34 (D), number of erythrocytes on D-10 (E) and D+34 (F) and hemoglobin concentration on D-10 (G) and D+34 (H). ^+^ Statistical tendency (0.05 < *P* < 0.1) in Tukey’s test; ^*^ Statistical significance (*P* < 0.05) in Tukey’s test.

On D-10, NPG mean hemoglobin concentration was higher (*P* = 0.04), while hematocrit and erythrocyte tended to be higher (*P* = 0.06 and *P* = 0.09, respectively; Figure 1). Regarding comparisons of sampling period within groups, hematocrit and total plasmatic protein was higher on D+34, comparing to D-10, for both PG and NPG heifers (Table 2). However, for both groups, the hematological parameters were found to be within physiological range on D-10 and D+34, and no other differences were found. The higher hematocrit, hemoglobin and erythrocyte means for NPG on D-10 were probably due to individual physiological factors, given that hematological parameters are influenced by many factors such as breed, age, sex, seasonal variations, pregnancy, health status and nutrition status (Mohammed et al., 2007).

**Table 2:** Comparison between hematological parameters in different sampling periods within pregnant (PG) and non-pregnant (NPG) Angus heifers on the first day (D-10) and 34 days after insemination (D+34) of the timed artificial insemination protocol.

Parameters	PG ($\bar{X}\pm SE$)		*P*	NPG ($\bar{X}\pm SE$)		*P*
	D-10	D+34		D-10	D+34	
Hematocrit (%)	35.92±0.71	39.13±0.65	0.006^*^	37.25±0.63	39.81±0.64	0.0257*
Total plasma protein (g/dL)	7.65±0.08	8.03±0.07	0.0027^*^	7.64±0.07	8.14±0.07	<.0001^*^
Erythrocyte (× 10^6^/µL)	7.47±0.13	7.52±0.13	0.99	7.72±0.12	7.48±0.12	0.4898
Hemoglobin (g/dL)	12.07±0.21	12.54±0.22	0.4128	12.56±0.20	12.45±0.20	0.9809

* Statistical significance (*P* < 0.05) in Tukey’s test.

Reproductive hormones can increase total plasma protein, due to their anabolic effects (Eckersall, 2008), which would explain the higher means for plasma protein in both groups on D+34. Other causes for higher plasma protein may include the stress of handling for the TAI protocol and pregnancy diagnosis, which could promote an inflammatory reaction (Cooke et al., 2012); or increased intestinal absorption of protein, due to changes to the heifers’ diet (Law et al., 2009).

It's noteworthy that all heifers were positive for *A. marginale* and *B. bovis* in the first qPCR, therefore all samples were submitted to the second qPCR to determine *B. bigemina* and *B. bovis* infection levels. However, in the analysis on absolute quantification, six animals were negative for *B. bigemina* (four PG and two NPG) and two were negative for *B. bovis*, one from each group, which could be explained by the different qPCR conditions in the first and second evaluations. Maximum values of CN were 0.59 copies/µL for *B. bigemina* in a NPG heifer and 2,3856.16 copies/µL for *B. bovis* in a PG heifer. The CN for *B. bigemina* tended to be higher in NPG heifers (Table 3), however since hematological parameters were not affected, further studies on beef and dairy herds with history of clinical manifestation of babesiosis and anaplasmosis are necessary to enable better understanding of the influence of these chronic infections on animal efficiency and fertility.

**Table 3:** Number of DNA copies (CN) of *Babesia bigemina* and *B. bovis* (mean ± SE) in pregnant (PG) and non-pregnant (NPG) Angus heifers on the first day (D-10) of the fixed timed artificial insemination protocol.

Parameters	PG ($\bar{X}\pm SE$)	NPG ($\bar{X}\pm SE$)	*P*
CN *B. bigemina* (copies/μL)	0.015 ± 0.005	0.037 ± 0.16	0.068+
CN *B. bovis* (copies/μL)	2,413 ± 614.8	1,419 ± 333.4	0.11

+ Statistical tendency (0.05 < *P* < 0.1) in Wilcoxon’s test.

Significant positive correlation coefficients were found between hemoglobin/erythrocyte (r = 0.8082; *P* < 0.0001) and between hemoglobin/hematocrit (r = 0.3009; *P* = 0.0049), while hematocrit tended to correlate positively with erythrocyte (r = 0.1862; *P* = 0.086) (Table 4). No significant correlation was found between *B. bigemina* and *B. bovis* CN and hematological variables. In this study, it was not possible to perform a proper evaluation on tick count due to the handling procedures on the farm. However, in an investigation on *B. bovis* and *B. bigemina* in cows and calves of different beef breeds in the state of São Paulo, higher *B. bovis* NC was also detected, but no correlation was found between the number of ticks and the hosts’ parasitemia (Giglioti et al., 2016). In other correlation analyses, tick infection levels showed a positive correlation with the CN of these parasites, thus indicating that the variation of parasitemia levels in the host depends not only on the presence of ticks, but also on the infection load of the vectors (Giglioti et al., 2018) and on the host’s immunological response (Palmer & Clothier, 2015; Zaugg, 2015).

**Table 4:** Correlation matrix of hematological parameters and number of *Babesia bigemina* and *B. bovis* copies in Angus heifers before the timed artificial insemination.

**Variables**	**CN *B. bigemina***	**CN *B. bovis***	**Hematocrit**	**Total plasma protein**	**Erythrocyte**	**Hemoglobin**
**CN *B. bigemina***	1.000**^1^**	0.0773**^1^**	0.0691**^1^**	-0.1998^1^	0.0993**^1^**	0.0584**^1^**
**CN *B. bovis***		1.000**^1^**	-0.1318**^1^**	0.0561**^1^**	0.0832**^1^**	0.0403**^1^**
**Hematocrit**			1.000**^2^**	0.1221^2^	0.1862**^2^**+	0.3009^2^*
**Total plasma protein**				1.000**^2^**	0.1175**^2^**	0.1734**^2^**
**Erythrocyte**					1.000**^2^**	0.8082^2^**
**Hemoglobin**						1.000**^2^**

1 Spearman’s ρ;

2 Pearson’s r;

+ 0.05 < *P* < 0.1;

* *P* < 0.05;

** *P* < 0.0001.

No other studies examining the relationships of erythrocyte count and hemoglobin concentration with regard to qPCR data for *Babesia* spp. in Angus females were found. In an investigation on cattle of different breeds and ages, Bilhassi et al. (2014) only found a significant negative correlation (-0.29) between the CN of *B. bovis* and hematocrit in relation to Nellore cows, while the other coefficients were close to zero. In the present study, the coefficient between these same variables was -0.13, but was also considered insignificant. Even in cattle with anemia (hematocrit < 25%), no correlation was observed between hematocrit and the number of DNA copies of *A. marginale* (Ramos et al., 2019). Although no absolute quantification of *A. marginale* was performed in the present study, the data referring to the numbers of copies of *B*. *bigemina* and *B. bovis* also showed no significant correlation with the other hematological variables.

Although most of Brazil is considered endemic for TF agents, there are several areas of enzootic instability in the state of Rio Grande do Sul (Santos et al., 2019). Thus, persistently infected animals, identified through molecular techniques, are at risk of increased parasitemia and recurrence of acute infection, thus increasing the economic losses (Kocan et al., 2010). Pregnant females are at higher risk, considering that these parasites can be transmitted to the fetus, thus causing pregnancy loss and stillbirth (Henker et al., 2020). It is therefore important to determine whether prevention strategies for stressful periods is necessary for cows (Silva & Fonseca, 2014), according to the reality of each herd.

## Conclusions

Low levels of *A. marginale* and *Babesia* spp. did not affect hematological parameters of chronically infected pregnant and non-pregnant taurine heifers. The number of DNA copies for *B. bigemina and B. bovis* did not differ between groups and showed no significant correlations with hematocrit, erythrocyte count and hemoglobin concentration.

## Supplementary Material

Supplementary material accompanies this paper.

Supplementary file 1

This material is available as part of the online article from https://doi.org/10.1590/S1984-29612023052
