# Supplementary figures and images for: Association between chronic Anaplasma marginale and Babesia spp. infection and hematological parameters of taurine heifers
Source: Rev Bras Parasitol Vet. 2023 Sep 1;32(3):e006423. doi: 10.1590/S1984-29612023052 (PMC10503823; doi:10.1590/S1984-29612023052)

## Supplementary file 1

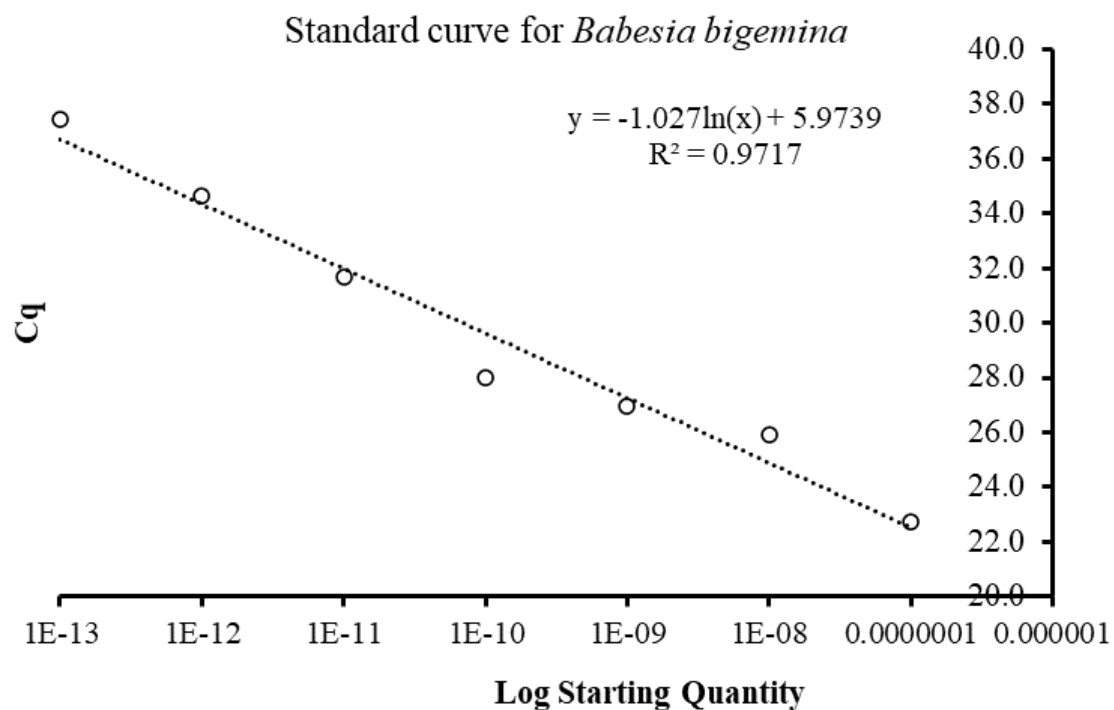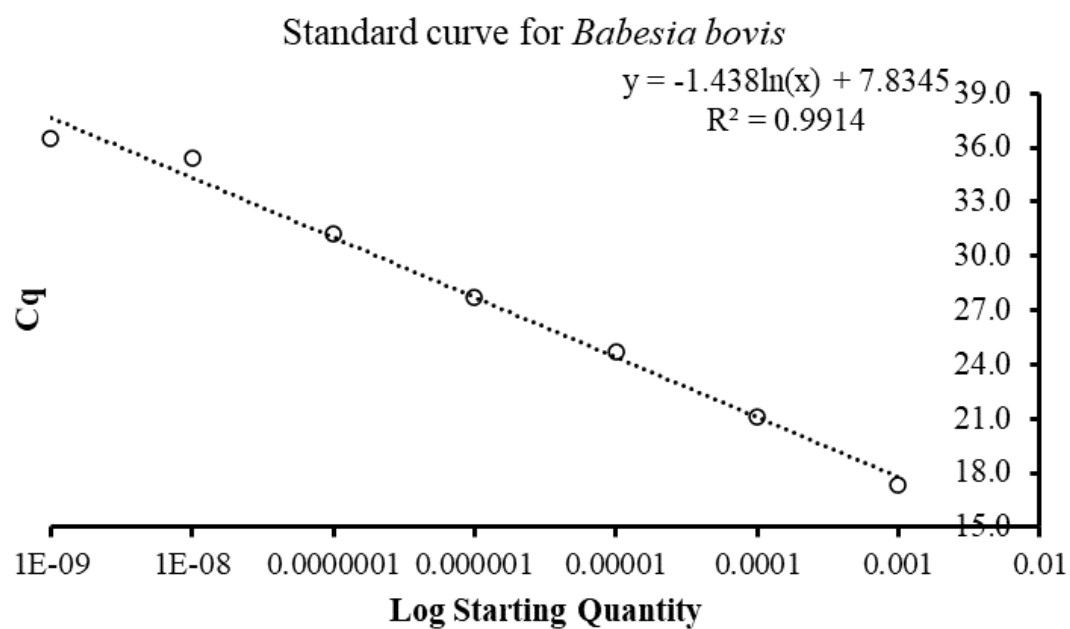

Supplement: Supplementary file 1 [file rbpv-32-3-e006423-supl.pdf]
